# Items

**Published:** 1901-08

**Authors:** 


### ITEMS.

INFANT feeding is an especially important and difficult problem during the heated term. The directions formulated by one of the leading American specialists, which were first published by The Maltine Company last summer, proved of immense service to many progressive practitioners. A new edition, comprising clear instructions and a liberal number of detachable recipes, tastefully bound, can be had gratis by applying to the Maltine Company, Eighth Avenue and Eighteenth Street, Brooklyn, N.Y.

THE M. J. Breitenbach Co., 53 Warren Street, New York City, representatives of Gude's pepto-mangan, most cordially invite all members of the medical profession when visiting New York City to make their office a business home.

MESSRS. REED & CARNRICK have just issued "Clinical Records, No. iii.," relating to the administration of protonuclein as a reconstructive agent. The records are in detail as to conditions and therapy, but names and addresses of patients and physicians are omitted. These can be supplied on request, in case of such desire, but this plan is commendable, relieving physicians of embarrassment in supplying such important data.

